# Goblet Cell Adenocarcinoma Presenting as Treatment-Resistant Ileocecal Stricture Mimicking Crohn’s Disease: Report of a Case

**DOI:** 10.70352/scrj.cr.26-0252

**Published:** 2026-07-29

**Authors:** Yasuki Sasaoka, Kota Arima, Yuji Miyamoto, Ayane Kawata, Takahiko Akiyama, Katsuhiro Ogawa, Yukiharu Hiyoshi, Masaaki Iwatsuki

**Affiliations:** Department of Gastroenterological Surgery, Graduate School of Medical Sciences, Kumamoto University, Kumamoto, Kumamoto, Japan

**Keywords:** goblet cell adenocarcinoma, Crohn’s disease, ileocecal stricture

## Abstract

**INTRODUCTION:**

Goblet cell adenocarcinoma (GCA) is a rare malignancy of the appendix that often involves deeper layers of the bowel wall, making preoperative diagnosis difficult and occasionally mimicking inflammatory bowel disease.

**CASE PRESENTATION:**

We report a 47-year-old man with a history of appendectomy who presented with an ileocecal stricture initially diagnosed as Crohn’s disease. As the stricture persisted despite repeated endoscopic balloon dilations, ileocecal resection was performed. Histopathological examination revealed that immunohistochemistry was positive for mucin 2, synaptophysin, and carcinoembryonic antigen, leading to a diagnosis of GCA. The patient received adjuvant chemotherapy with capecitabine and oxaliplatin and remains recurrence-free.

**CONCLUSIONS:**

This case indicates that malignancy should be considered in treatment-refractory ileocecal strictures, even when endoscopic biopsy results are negative.

## Abbreviations

CA19-9
: carbohydrate antigen 19-9
CEA
: carcinoembryonic antigen
GCA
: goblet cell adenocarcinoma
MUC2
: mucin 2

## INTRODUCTION

Crohn’s disease is an intractable inflammatory disease that can cause inflammation and ulceration in any part of the gastrointestinal tract.^1,2)^ When complications such as strictures, perforations, fistulas, or abscess formation occur, surgical intervention becomes necessary.^1,2)^ We herein report a case in which surgery was performed for an ileocecal stricture presumed to be caused by Crohn’s disease; however, the final diagnosis revealed GCA originating from the colon.

## CASE PRESENTATION

**Fig. 1:**
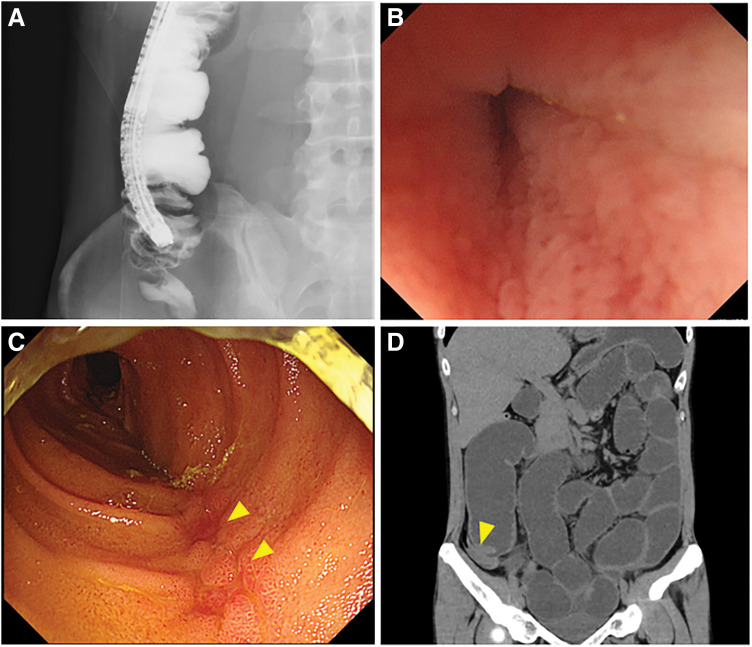
Preoperative findings. (**A**) Site of stenosis identified by gastrointestinal contrast imaging. (**B**) Severe stenosis at the ileocecal region. (**C**) Longitudinal ulcer in the terminal ill proximal to the stenotic segment (arrowheads). (**D**) Preoperative CT scan of severe stenosis (arrowhead).

A 47-year-old man with a history of appendectomy for acute appendicitis at another hospital at the age of 35 was referred to our hospital. He had also been followed by the Department of Nephrology at our hospital for minimal change nephrotic syndrome since the age of 43. Two years prior, abdominal CT had revealed stenosis at the terminal ileum and ileocecal region. Laboratory findings revealed mild leukocytosis, with no other abnormal findings, and no elevation of serum CEA and CA19-9. A gastrointestinal contrast study also demonstrated stenosis at the same site (**Fig. 1A**). Colonoscopy demonstrated a longitudinal ulcer proximal to the stenotic segment (**Fig. 1B** and **1C**). Biopsy specimens obtained from the ileocecal region showed only nonspecific inflammatory changes in the lamina propria without evidence of malignancy, and he was diagnosed with Crohn’s disease. At that time, the patient had already been receiving oral corticosteroid therapy and cyclosporine for minimal change nephrotic syndrome under the care of the nephrologist. For Crohn’s disease, only nutritional therapy was performed.

Endoscopic balloon dilation was performed 5 times for the stenotic lesion. However, severe stenosis persisted despite repeated dilations (**Fig. 1D**); thus, surgical resection was planned for definitive management. The ileocecal segment was mobilized from the retroperitoneum using a laparoscopic approach and exteriorized through a small midline incision. The ileocolic artery and vein were divided at their roots. As a firm, indurated lesion corresponding to the stenosis was identified, ileocecal resection, including a dilated and edematous bowel segment, was performed. Oral intake was resumed on POD 2, and the patient was discharged on POD 11 without any complications.

**Fig. 2:**
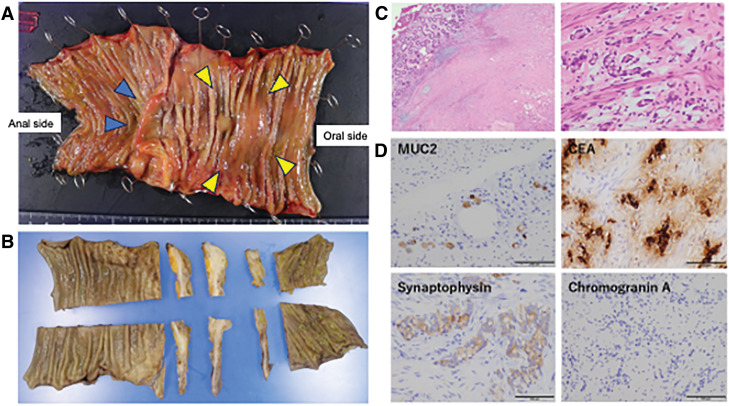
Resected specimen. (**A**) Fresh appearance of the ileocecal segment showing wall thickening and sclerosis at the stenotic site. The blue arrowheads represent the ileocecal valve, and the yellow arrowheads represent the tumor. (**B**) Formalin-fixed specimen demonstrating the same stenotic lesion with marked mural thickening. (**C**) Left: Low-power view (×100) showing tumor infiltration in the muscularis propria with preserved mucosa. Right: High-power view (×400) showing mucin-containing tumor cells. (**D**) Immunohistochemistry showing positivity for MUC2, CEA, and synaptophysin, and negativity for chromogranin A. CEA, carcinoembryonic antigen; MUC2, mucin 2

Macroscopic findings showed a thick and sclerotic ileocecal segment wall (**Fig. 2A** and **2B**), and histological findings showed invasive tumor cells with abundant intracellular mucin predominantly involving the muscularis propria and extending to the serosa (**Fig. 2C**). Immunohistochemical findings showed the tumor cells were positive for MUC2, synaptophysin, and CEA, and negative for chromogranin A (**Fig. 2D**). Based on these findings, he was diagnosed with GCA (pT4aN1b, Stage IIIb). Histopathological examination demonstrated no lymphovascular invasion, high-grade tumor budding (BD3), and the presence of perineural invasion (Pn1b). Both the proximal and distal resection margins were negative. Adjuvant chemotherapy with capecitabine and oxaliplatin was administered for 4 cycles because of positive lymph node metastasis. The patient is currently undergoing follow-up with tumor markers every 3 months and CT every 6 months, and no recurrence has been detected to date. Following the pathological diagnosis of GCA, no further treatment for Crohn’s disease was required. The patient has continued treatment for minimal change nephrotic syndrome under the care of the nephrologist.

## DISCUSSION

GCA was previously classified as a subtype of neuroendocrine tumor and referred to as carcinoid or adenocarcinoid. However, in the World Health Organization Classification of Digestive System Tumours, 5th edition, it was redefined as a distinct subtype of adenocarcinoma arising in the appendix.^3)^ GCA is a rare malignancy, with an estimated incidence of approximately 0.05–0.3 cases per 100000 population.^2)^ Although the number of reported cases has increased in recent years due to improved disease recognition and pathological classification, it remains an uncommon entity.^4,5)^

One of the clinical characteristics of GCA is the difficulty of diagnosing preoperatively. Many cases are initially treated as acute appendicitis, chronic inflammation, or ileocecal stenosis, and the diagnosis is established just only after pathological examination of the resected specimen.^6)^ GCA frequently involves the muscularis propria and serosa, with minimal or secondary mucosal involvement, which may result in false-negative endoscopic biopsies.^7)^ In cases such as ours, where mucosal biopsies show only nonspecific inflammatory changes, differentiation between colitis-associated carcinoma and incidental malignancy is often challenging in patients with inflammatory bowel disease. In the present case, re-evaluation by an experienced pathologist showed no characteristic histological features suggestive of colitis-associated carcinoma. Therefore, this lesion was considered more likely to represent an incidental GCA. Nevertheless, because the tumor predominantly involved the deeper layers of the bowel wall and only minimally affected the mucosa, preoperative distinction from Crohn’s disease was difficult. Negative endoscopic biopsy findings and clinical features suggestive of Crohn's disease initially favored a benign diagnosis. However, persistent stenosis despite repeated endoscopic balloon dilations should raise suspicion of underlying malignancy. This case highlights the importance of considering neoplastic disease in patients with treatment-resistant Crohn-like strictures.

The primary site of the tumor could not be definitively determined. Although most reported GCAs arise from the appendix, pathological re-evaluation of the previously resected appendix was impossible because the specimen had already been discarded. Therefore, while an appendiceal origin is considered more likely, a primary lesion arising from the ileocecal region cannot be completely excluded. Also, late recurrence from an appendiceal primary cannot be excluded. Several mechanisms should be considered in this case. These include late recurrence from an occult appendiceal GCA with hematogenous or peritoneal dissemination, as well as local recurrence arising from residual appendiceal tissue or the appendiceal stump. Because pathological assessment of the original appendectomy specimen was unavailable, these possibilities could not be definitively evaluated. On the other hand, sporadic reports have described GCA arising outside the appendix, including in other segments of the gastrointestinal tract.^8–10)^ Although true extra-appendiceal primary GCA is exceedingly rare and requires careful exclusion of an appendiceal origin, the possibility of small intestinal or ileocecal origin cannot be completely ruled out in this case.

A review of previously reported cases revealed that GCA frequently poses diagnostic challenges because of its tendency to infiltrate the deeper layers of the bowel wall with minimal mucosal involvement (**Table 1**).^11–13)^ Consequently, false-negative endoscopic biopsy results are not uncommon, emphasizing the importance of maintaining a high index of suspicion in atypical or treatment-resistant cases.

**Table 1:** Summary of previously reported cases of GCA

Author	Year	Initial diagnosis	Diagnostic pitfall	Treatment before definitive diagnosis	Final diagnosis
Lenti et al.^11)^	2021	Crohn’s disease	Inflammatory ileal stricture with repeatedly negative biopsies mimicked Crohn’s disease	Medical therapy for Crohn’s disease	Primary appendiceal-type GCA of the ileum
Sigley et al.^12)^	2021	Acute appendicitis	GCA diagnosed only after histopathological examination following appendectomy	Laparoscopic appendectomy	Appendiceal GCA
Fukasawa et al.^13)^	2024	Ovarian tumor	Ovarian mass was initially considered a primary ovarian neoplasm	Surgical resection for presumed ovarian tumor	Appendiceal GCA with ovarian metastasis
Present case	2026	Crohn’s disease	Long-segment ileocecal stricture with repeated negative biopsies mimicked Crohn’s disease	Endoscopic balloon dilation ×5	GCA (pT4aN1b)

GCA, goblet cell adenocarcinoma

With regard to treatment, GCA with lymph node metastasis is generally managed according to colon adenocarcinoma treatment paradigms. Although high-level evidence specific to GCA is lacking, several reports suggest that adjuvant chemotherapy following colon cancer protocols is appropriate. In colon cancer, a 6-month duration of adjuvant chemotherapy is generally recommended for high-risk disease stage, such as T4 tumors.^14)^ In the present case, adjuvant chemotherapy with capecitabine and oxaliplatin was administered. The duration of adjuvant chemotherapy was limited to 3 months because of impaired renal function associated with minimal change nephrotic syndrome. The patient remains disease-free at the time of this report.

## CONCLUSIONS

This case underscores the importance of considering GCA in patients with Crohn-like, treatment-resistant ileocecal strictures. Even in the presence of negative biopsies, persistent or atypical stenotic lesions should be performed with careful and multidisciplinary evaluation, including surgical intervention.

## DECLARATIONS

### Availability of data and material

All data generated or analyzed during this study are included in the published article.
